# Enhanced efficacy and limited systemic cytokine exposure with membrane-anchored interleukin-12 T-cell therapy in murine tumor models

**DOI:** 10.1136/jitc-2019-000210

**Published:** 2020-01-19

**Authors:** Ling Zhang, John S Davies, Carylinda Serna, Zhiya Yu, Nicholas P Restifo, Steven A Rosenberg, Richard A Morgan, Christian S Hinrichs

**Affiliations:** 1 Experimental Transplantation and Immunology Branch, Center for Cancer Research, National Institutes of Health, Bethesda, Maryland, USA; 2 Surgery Branch, Center for Cancer Research, National Institutes of Health, Bethesda, Maryland, USA; 3 Lyell Immunopharma, South San Francisco, California, USA; 4 Immunogenetics, Editas Medicine, Cambridge, Massachusetts, USA

**Keywords:** immunology, tumours

## Abstract

**Background:**

Interleukin-12 (IL-12) is a potent, proinflammatory cytokine that holds promise for cancer immunotherapy, but its clinical use has been limited by its toxicity. To minimize systemic exposure and potential toxicity while maintaining the beneficial effects of IL-12, we developed a novel IL-12-based therapeutic system that combines tumor-specific T-cell-mediated delivery of IL-12 with membrane-restricted IL-12 localization and inducible IL-12 expression.

**Methods:**

Therapeutic T cells targeting a tumor antigen were genetically engineered to express membrane-anchored IL-12 (aIL-12). Expression, function, and shedding of the aIL-12 molecule was assessed in vitro. Tumor treatment efficacy was assessed in vivo with T cell receptor (TCR) transgenic murine tumor models and a tumor xenograft model. Key outcomes were change in tumor size, circulating levels of IL-12 and other cytokines, and survival. Toxicity was assessed via change in body weight. Tumor growth curve measurements were compared using repeated-measures two-way analyses of variance.

**Results:**

Retroviral gene transfer resulted in cell membrane expression of aIL-12 by transduced T cells. In each of two transgenic murine tumor models, tumor-specific T cells constitutively expressing aIL-12 demonstrated increased antitumor efficacy, low circulating IL-12 and interferon-γ, and no weight loss. Expression of aIL-12 via a *NFAT-*inducible promoter resulted in coordinate expression of aIL-12 with T cell activation. In an OT-I TCR transgenic murine tumor model, the *NFAT*-inducible aIL-12 molecule improved tumor treatment and did not result in detectable levels of IL-12 in serum or in weight loss. In a human tumor xenograft model, the *NFAT*-inducible aIL-12 molecule improved antitumor responses by human T cells coexpressing a tumor-specific engineered TCR. Serum IL-12 levels were undetectable with the *NFAT*-inducible construct in both models.

**Conclusion:**

Expression of aIL-12 by tumor-targeting therapeutic T cells demonstrated low systemic exposure and improved efficacy. This treatment strategy may have broad applications to cellular therapy with tumor-infiltrating lymphocytes, chimeric antigen receptor T cells, and TCR T cells.

## Background

T-cell therapy is emerging as a cancer therapy that may hold promise for treating a wide range of cancers.^1 2^ Chimeric antigen receptor T cells have demonstrated efficacy in B-cell leukemias and lymphomas. Clinical activity has been reported with tumor-infiltrating lymphocytes for melanoma^3^ and for human papillomavirus (HPV)-associated epithelial cancers.^4–6^ T cell receptor genetically engineered T cells (TCR-T cells) have shown clinical activity in melanoma,^7^ sarcoma,^7^ and HPV-associated epithelial cancers.^8^ Nonetheless, despite remarkable tumor responses in patients with these cancers and other epithelial cancers,^9 10^ enhanced efficacy remains an important goal in the development of cellular therapies.

One strategy to increase the efficacy of cellular therapy is to combine administration of tumor-specific T cells with interleukin-12 (IL-12), a potent activator of innate and adaptive immunity.^11 12^ IL-12 is a heterodimeric protein composed of a 35 kDa light chain (p35 or IL-12α) and a 40 kDa heavy chain (p40 or IL-12β) that is mainly produced by phagocytes and dendritic cells. IL-12 primarily acts on natural killer cells and T cells and induces T cells to acquire a type 1 differentiation profile characterized by an increased production of interferon-γ (IFN-γ). The potential for IL-12 to induce antitumor immune responses has been demonstrated in a wide array of mouse models.^11 12^

In humans, systemic administration of recombinant human IL-12 as a single agent has resulted in severe toxicity.^13^ IL-12 delivery by genetically engineered tumor-specific T cells has been investigated as an alternative strategy to systemic infusion. In a clinical trial for the treatment of melanoma, autologous tumor-infiltrating lymphocytes that were genetically engineered to secrete IL-12 were administered to patients.^14^ To preferentially localize IL-12 to the tumor, IL-12 transcription was driven by a TCR-activated nuclear factor of activated T cells (NFAT) transcriptional response element promoter. Clinical activity was observed with a relatively low dose of therapeutic T cells; however, high serum levels of IL-12 and IFN-γ were noted, and severe IL-12-related toxicity limited further development of this strategy.

In this report, we describe a next-generation system to deliver IL-12 to tumors while limiting systemic exposure by expressing IL-12 on the membrane of therapeutic T cells using a transmembrane (TM) anchor domain. The efficacy of this approach and the systemic exposure to IL-12 and other cytokines were investigated using distinct in vivo tumor models with varied target antigens and with both murine and human T cells.

## Methods

### Construction of anchored IL-12 vectors

IL-12 constructs were generated with constitutive activity under a long-terminal repeat (LTR) promoter or inducible activity under an NFAT promoter. Constructs were named with a ‘c’ or ‘i’ prefix to denote constitutive (LTR promoter) versus inducible (NFAT promoter) expression and an ‘s’ or ‘a’ prefix to denote secreted versus membrane-anchored IL-12. Mouse single-chain IL-12 (mflexiIL12) was designed by linking p40 and p35 with a flexible linker (G_4_S)_3_ and cloned into MSGV1 retroviral vector to generate vector constitutive secreted IL-12 (csIL-12).^15^ Inducible secreted IL-12 (isIL-12) was generated by inserting NFAT-mfllexiIL12 cassette into MSGV1 vector in a direction against the 5’ LTR as previously described.^16^ Mouse B7-1, CD28 or CD8 TM domains were synthesized by Epoch Biolab (Sugar Land, Texas, USA) and cloned in-frame downstream of mflexiIL12 to generate constitutive anchored IL-12.B7 (caIL-12.B7), caIL-12.CD8 and caIL-12.CD28. The sequences of the B7, CD8, and CD28 TM domains are listed in online supplementary table S1. The expression cassette of IL-12.B7 was also cloned into the self-inactivating retroviral vector SERS11NFAT.GFP to regulate the aIL-12 under the NFAT-responsive promoter, referred to as inducible anchored IL-12 (iaIL-12).

10.1136/jitc-2019-000210.supp1 Supplementary data

The human single-chain IL-12 vectors csIL-12 and isIL-12 were constructed in our laboratory.^17^ The human B7-1 TM domain was synthesized by GenScript (Piscataway, New Jersey, USA) and cloned downstream of single-chain IL-12 in the vector to generate caIL-12. IL-12.B7 was cloned into the self-inactivating retroviral vector SERS11NFAT.GFP under an NFAT-responsive promoter to generate human iaIL-12.

### Mice and tumor cell lines

Pmel-1 (gp100) TCR and OT-I transgenic mice were obtained from the Jackson Laboratory (Bar Harbor, Maine, USA) and housed at the National Institutes of Health (Bethesda, Maryland, USA). Splenocytes from pmel-1, OT-I, and P14 transgenic mice were used as donors for adoptive cell therapy. All experiments were conducted with the approval of the National Cancer Institute Animal Care and Use Committee.

The B16 tumor cell line (Cat. No. CRL-6475, ATCC, Manassas, Virginia, USA) was used for in vivo experiments. The B16-OVA line was established by transducing B16 cells with a retrovirus expressing the mKate2-SIINFEKL fusion protein. Transduced cells were then sorted by mKate2 fluorescence using a BD influx cell sorter (BD Biosciences, San Jose, California, USA). The cell line SS4050 is an HPV-16^+^ human leucocyte antigen (HLA)-A*0201^+^ cervical cancer line that was generated in our laboratory from a lung metastasis. CaSki cells were purchased from ATCC (Cat. No. CRM-CRL-1550). The melanoma cell line 624 was generated previously by the Surgery Branch of the National Cancer Institute (Bethesda, Maryland, USA). All tumor cell lines were cultured using Dulbecco Modified Eagle Medium with 10% heat-inactivated fetal bovine serum (Gemini Bio-Products, Sacramento, California, USA) at 37°C in an atmosphere containing 5% CO_2_.

### Transient retroviral vector preparation and T-cell transduction

To produce the γ-retrovirus, package cell line 293GP or Platinum Eco cells (Cat. No. RV-101, Cell Biolabs, San Diego, California, USA) were cotransfected with 9 µg of target vector DNA and 4 µg envelope plasmid (Rd114 envelope was used to produce virus to infect human T cells; pEco envelope was used to produce virus to infect murine T cells) using lipofectamine 2000 (Cat. No. 11668019, Invitrogen, Carlsbad, California, USA) on a 100 mm^2^ poly-D-lysine–coated plate (Corning, New York, USA). Viral supernatants were harvested 48 and 72 hours after transfection.

For mouse T-cell transduction, splenocytes derived from TCR transgenic mice (pmel-1 or OT-I) were harvested and stimulated with 2 µg/mL plate-bound anti-mouse CD3 antibody (Clone 145–2 C11, Cat. No. BE0001-1, Bio X Cell, Lebanon, New Hampshire, USA) and 1 µg/mL anti-mouse CD28 antibody (Clone 37.51, Cat. No. 37.51, Bio X Cell) in RPMI medium supplemented with 10% fetal bovine serum and 60 IU/mL interleukin-2 (IL-2; Novartis, Basel, Switzerland), and incubated at 37°C in an atmosphere containing 5% CO_2_. Two days later, the splenocytes were harvested and transduced with γ-retroviral vectors using a RetroNectin (Cat. No. T202, Takara Bio, Kusatsu, Shiga Prefecture, Japan) coated plate as described previously.

For human T-cell transduction, human peripheral blood mononuclear cells were activated with 50 ng/mL OKT3 (Cat. No. 130-093-387, Miltenyi Biotec, Bergisch Gladbach, Germany) and harvested for retroviral transduction on day 2. Cells applied to vector-preloaded RetroNectin (Takara) coated non-tissue culture 6-well plates (Corning) at a concentration of 1×10^6^ per well and centrifuged at 1500rpm at 32°C for 10 minutes. After centrifugation, the cells were then cultured in AIM-V medium containing 5% human AB serum (Cat. No HP1022HI, Valley Biomedical, Winchester, Virginia, USA) and 300 IU/mL IL-2 until use.

### Adoptive cell transfer

C57BL/6 mice at 6–12 weeks of age were injected subcutaneously with 1×10^6^ B16 or B16-OVA tumor cells. Seven days later, tumor-bearing mice (n=5) were treated with 550 cGy whole-body irradiation and then underwent adoptive T-cell transfer by intraperitoneal or tail-vein injection. Tumors were measured using digital calipers twice a week. Serum from mice receiving T-cell transfer was collected at different time points after T-cell transfer. To measure cytokines in mouse serum, approximately 60 µL of mouse blood was drawn into Z-gel microtubes (Thermo-Fisher Scientific) by puncturing the submandibular vein at days 3, 10, or 14 and centrifuged at 12 000 rpm for 10 min. The serum was transferred to a 1.5 mL Eppendorf tube and saved at −20°C until multiple cytokine assays were performed using a customized V-Plex kit (Meso Scale Diagnostics). All experiments were performed independently at least twice, and all tumor curve data are shown as mean±SEM.

### Cytokine assay

Peripheral blood lymphocytes cultures were tested for reactivity in cytokine release assays using commercially available ELISA kits (ELISA; human IL-12 (Cat. No. S1200), IL-2 (Cat. No. S2050), IFN-g (Cat. No. SIF50), TNF-a (Cat. No. STA00D); R&D Biosystems, Minneapolis, Minnesota, USA). For these assays, 1×10^5^ responder cells (transduced T cells) and 1×10^5^ target cells (tumor cells) were incubated overnight (~16 hours) in a 0.2 mL culture volume in a 96-well plate. The supernatant was then collected and transferred into a new 96-well plate and saved for future ELISA assay at −20°C. Within a week, the supernatant was thawed and diluted to proper linear range to detect cytokines including IFN-γ, IL-2, and tumor necrosis factor-α (TNF-α) based on the manufacturer instruction. For T cells transduced by isIL-12, transduced T cells were activated with a cell stimulation cocktail containing phorbol 12-myristate 13-acetate (PMA) and ionomycin (Thermo-Fisher Scientific, Waltham, Massachusetts, USA) overnight to activate the NFAT-responsive promoter. IL-12 levels were examined in the culture using an ELISA. To measure cytokines in mouse serum, approximately 60 µL of mouse blood was drawn into Z-gel microtubes (Thermo-Fisher Scientific) at days 3, 10, or 14 and centrifuged at 12 000 rpm for 10 min. The serum was separated and saved for multiple cytokine assays using a customized V-Plex kit (Meso Scale Diagnostics).

### Flow-cytometry analysis

To detect IL-12 expression on the cell surface, IL-12-modified cells were harvested 2 days after transduction and stained with PE-conjugated anti-mouse IL-12 (Clone REA285, Cat. No. 130-103-821, Miltenyi Biotec) or allophycocyanin-conjugated antihuman IL-12 (Clone REA123, Cat. No. 130-103-738, Miltenyi Biotec). For inducible IL-12–transduced cells, cells were pretreated with a cell stimulation cocktail (Cat. No. 00-4970-93, Thermo-Fisher Scientific) overnight before analysis by flow cytometry.

### Statistical analysis

All analyses were performed with Prism7 (GraphPad Software, San Diego, California, USA). Tumor growth curve measurements were compared using repeated-measures two-way analyses of variance (ANOVAs). The Mantel-Cox test was used for survival curves. Two-way multiple-comparison ANOVA was used to test the significant differences in enumeration assays. P<0.05 was considered to be statistically significant.

## Results

### IL-12 was anchored to the T-cell membrane by fusion with a B7-1 TM anchor

**Figure 1:**
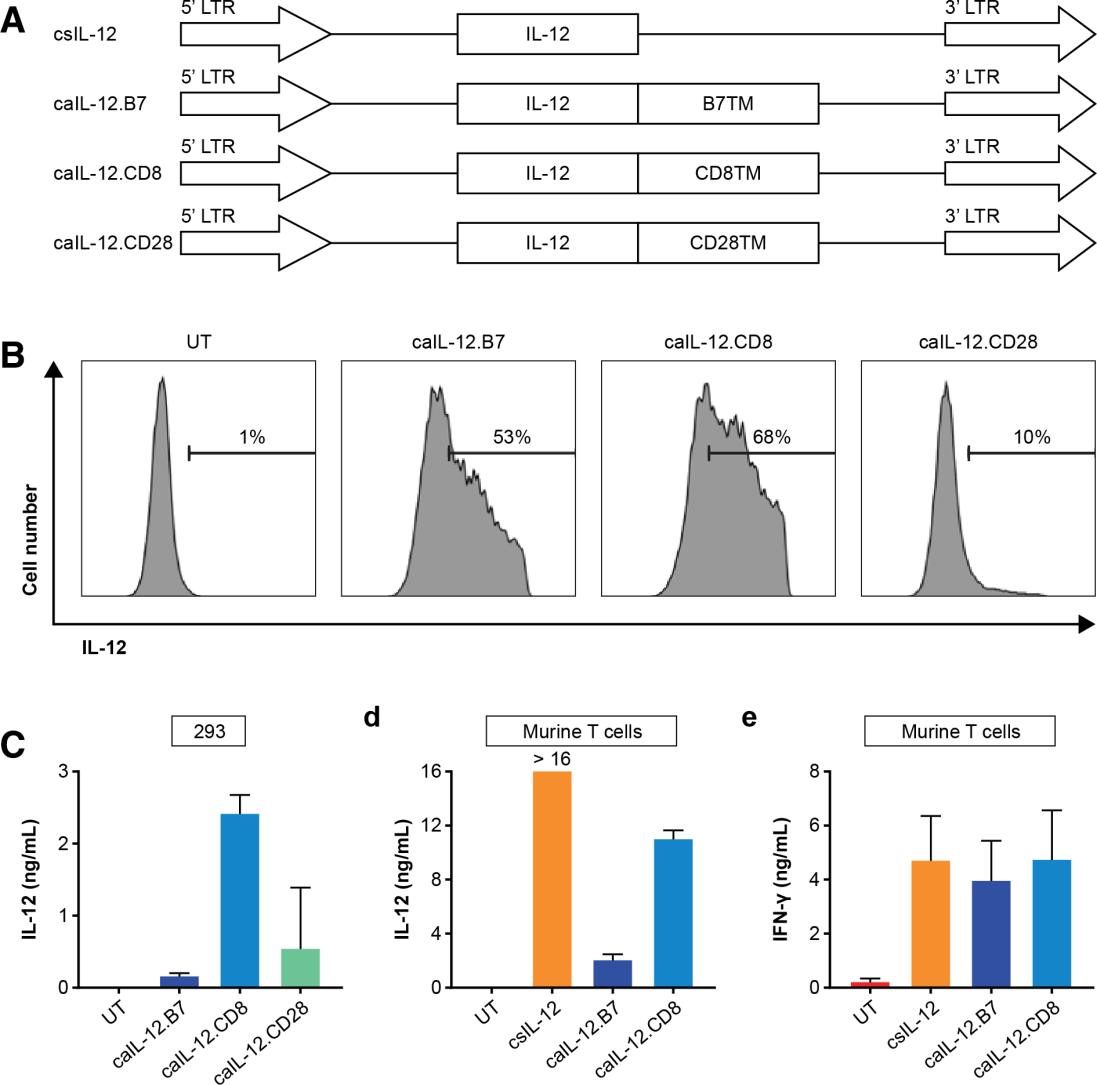
Optimization of caIL-12 constructs. (A) Schematic representation of retroviral vector inserts for expression of designer murine IL-12 constructs. (B) 293cells were transfected with vectors encoding IL-12 fused with different transmembrane domains. IL-12 on surface of the cell membrane was measured by flow cytometry performed 48 hours after transfection. Gating is on live cells. (C) The IL-12 level in the supernatant of transfected 293 cells was measured by ELISA 48 hours after transfection with the construct indicated on the x-axis. (D, E) Murine T cells from C57BL/6 mice were transduced with MSGV1-based retroviruses to express the constructs indicated on the x-axis. Approximately 48 hours after transduction, 1×10^6^ genetically modified T cells were incubated in 1 mL of culture medium overnight. The levels of (D) mouse IL-12 and (E) mouse IFN-γ in the culture were measured by ELISA. caIL-12, constitutive anchored interleukin-12; csIL-12, constitutively expressed secreted interleukin-12; IFN-γ, interferon-γ; IL-12, interleukin-12;; LTR, long-terminal repeat; UT, untransduced cells.

To enable expression of IL-12 from a single gene, an IL-12 molecule in which the p35 and p40 subunits are connected by a flexible linker was used.^18^ Three TM anchors for IL-12 were tested: B7-1, CD8, and CD28. Codon-optimized inserts were cloned into MSGV1 vectors (figure 1A). 293-Based cells were transfected to express IL-12 constructs possessing different anchor domains. IL-12 constructs with the B7-1 TM or CD8 TM, but not with the CD28 TM, were detected at high levels on the cell surface (figure 1B). However, high levels of IL-12 were detected in the supernatants of cell cultures with the CD8 TM but not the B7-1 TM (figure 1C). Similar soluble IL-12 results were also observed with murine T cells retrovirally transduced with IL-12 constructs. The constitutively expressed (LTR promoter), secreted IL-12 construct, csIL-12, served as a positive control. The culture supernatant of T cells expressing the B7-1 TM construct had lower soluble IL-12 than the culture supernatant of T cells expressing the CD8 TM construct (figure 1D). Importantly, expression of IL-12 via either the secreted or anchored constructs resulted in increased IFN-γ production by T cells, and anchoring IL-12 to the T-cell membrane did not reduce this effect (figure 1E). Although IL-12 in the culture supernatants was reduced by the B7-1 TM anchor, it was not completely eliminated, and some IFN-γ production may be due to soluble IL-12 shedding from the cell membrane. Because of its relatively high membrane expression and relatively low shedding, the IL-12 construct with a B7-1 TM, aIL-12, was selected for further study.

### Constitutive expression of aIL-12 improved tumor treatment in murine tumor models

**Figure 2:**
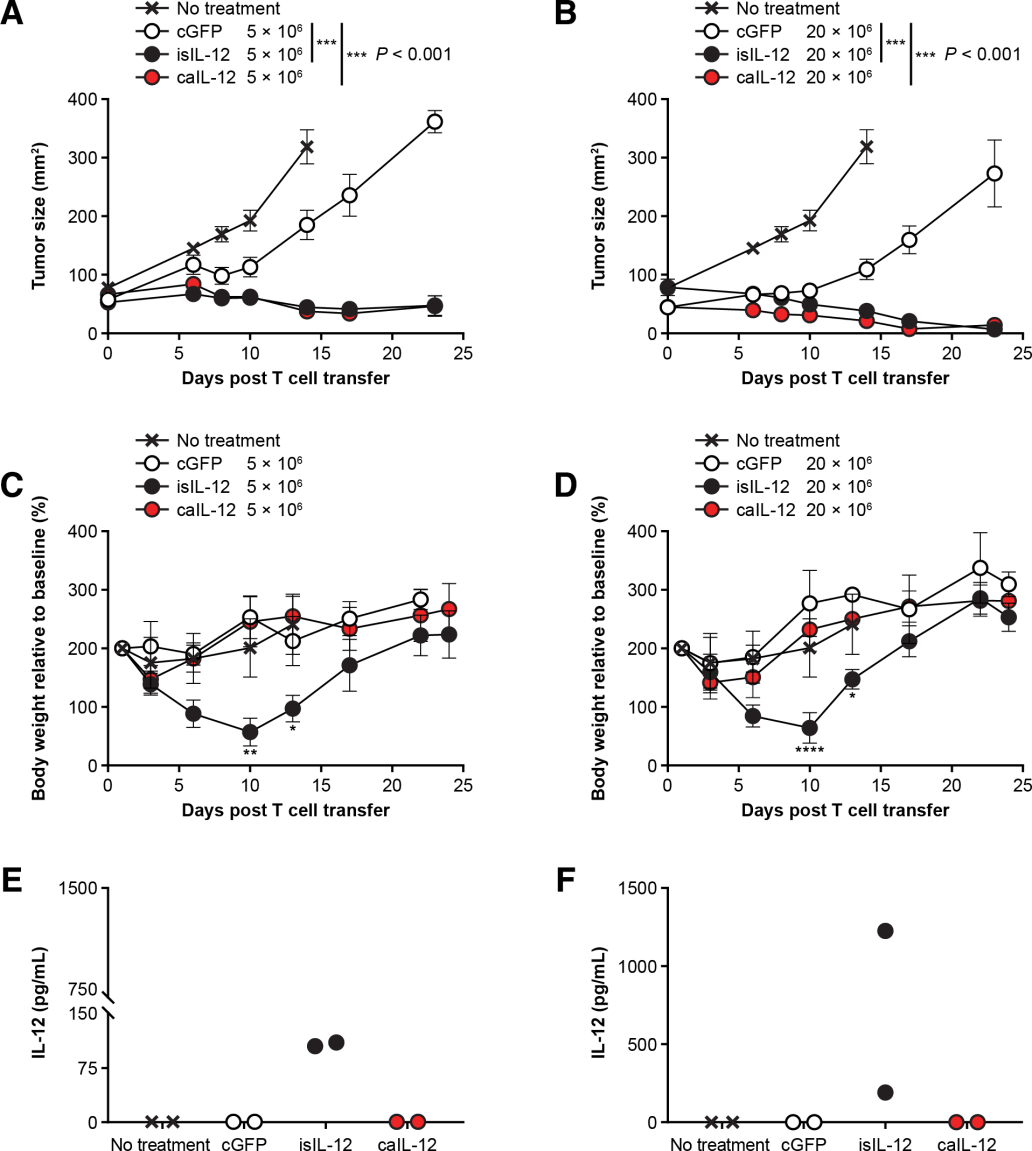
Pmel-1 T cells expressing caIL-12 displayed enhanced tumor regression without evidence of toxicity. (A, B) Pmel-1 T cells were transduced with the vectors indicated. Either (A) 5×10^6^ or (B) 20×10^6^ cells were administered by tail-vein injection to B16 tumor-bearing mice. All mice received 550 cGy of whole-body radiation before treatment. isIL-12 served as a positive control for systemic IL-12 exposure. Serial tumor measurements were obtained. N=5 mice per group. Error bars reflect the standard error of the mean. P values represent comparison between caIL-12 or isIL-12 and cGFP using a matched-pairs, repeated-measures two-way ANOVA test. (C, D) The change in body weight relative to baseline is shown. Error bars show the standard error of the mean. P values represent comparison between caIL-12 and isIL-12 at each time point using a two-way ANOVA multiple comparisons test. *P<0.05, **P<0.01, ****P<0.0001. (E, F) Serum IL-12 levels were determined for mice in panels A and B on day 3 after treatment. Values for individual mice are shown.; ANOVA, analysis of variance; caIL-12, constitutive anchored interleukin-12; cGFP, constitutively expressed green fluorescent protein; isIL-12, inducible secreted interleukin-12.

To determine if tumor-specific T cells expressing caIL-12 could augment tumor regression, pmel-1 TCR transgenic T cells, which target mouse gp100, were transduced with caIL-12 and administered to B16 tumor-bearing mice. A dose-dependent antitumor response was noted (online supplementary figure S1). The antitumor responses occurred in the absence of adjuvant gp100 vaccine and systemic IL-2, which were not administered in this experiment but were required in the original report of tumor regression in this TCR transgenic model.^19^ To test if caIL-12 enhances the efficacy of antitumor T-cells, we compared tumor treatment with pmel-1 cells expressing caIL-12 to treatment with pmel-1 cells expressing green fluorescent protein. The previously studied isIL-12 vector (analogous to the human IL-12 vector used in clinical trials) served as a positive control for IL-12 efficacy and IL-12 systemic exposure. Pmel-1 cells with caIL-12 induced significantly enhanced tumor regression (p<0.001, figure 2A, B). Furthermore, in contrast to the isIL-12 vector, the caIL-12 vector did not result in toxicity, as measured by weight loss, compared with the other groups (figure 2C, D). Finally, systemic exposure to IL-12 was assessed by serum levels determined 3 days after treatment. Elevated serum levels of IL-12 were not detected in mice that received the caIL-12 construct (figure 2E, F). The findings of enhanced tumor treatment, an absence of weight loss and undetectable levels of serum IL-12 with the caIL-12 vector were observed at both doses of pmel-1 cells that were tested (5×10^6^ and 20×10^6^ cells) and aIL-12 improved tumor treatment in a dose-dependent manner (online supplementary figure S1).

**Figure 3:**
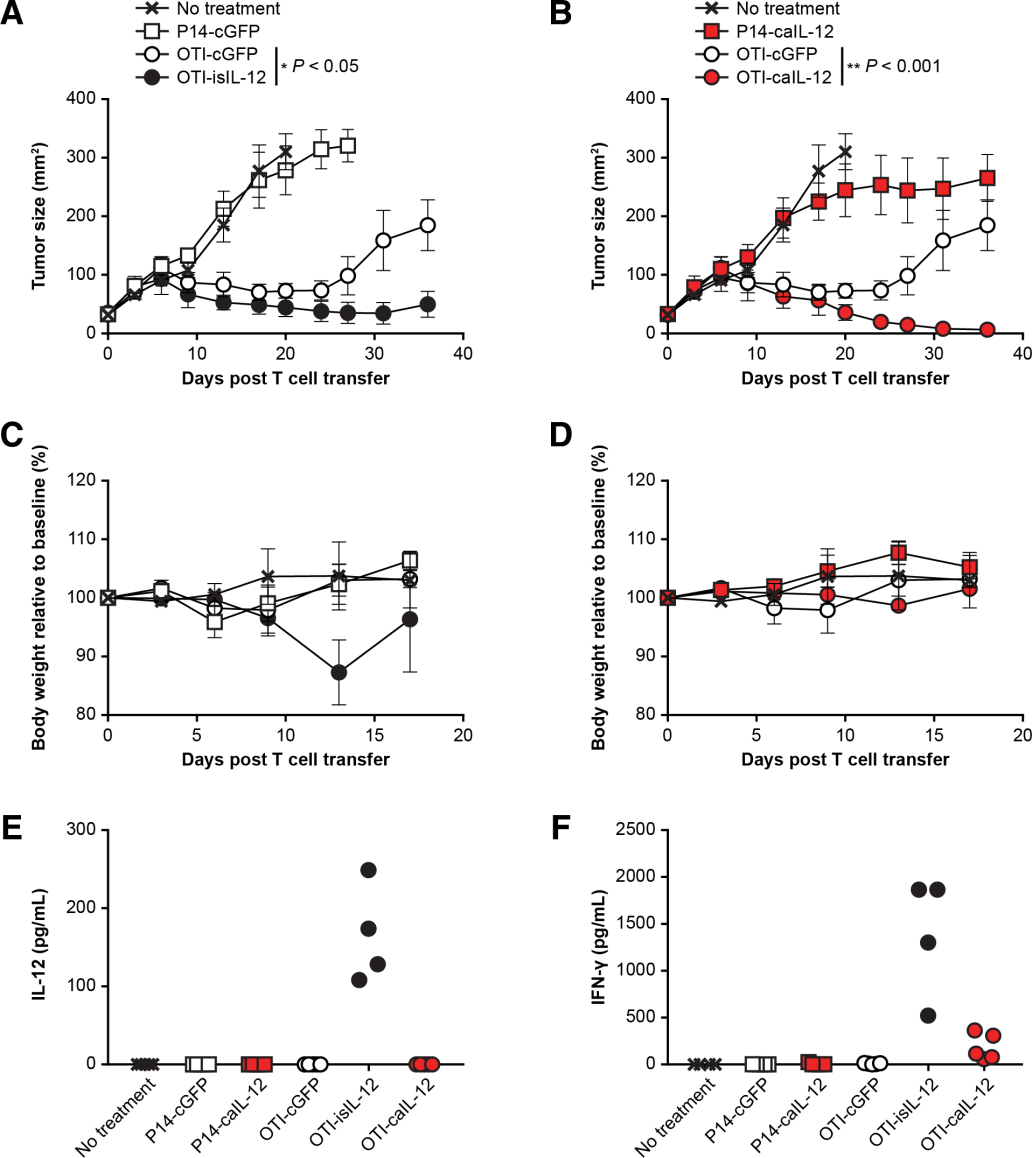
OT-I T cells expressing caIL-12 displayed enhanced tumor regression without evidence of toxicity. OT-I or P14 transgenic T cells transduced to express the constructs indicated in the figure legends and were administered to mice bearing established, subcutaneous B16-OVA tumors. All mice received 550-cGy whole-body irradiation. Cells (5×10^6^) were administered by intraperitoneal injection. isIL-12 served as a positive control for systemic IL-12 exposure. Data shown are from one experiment that was independently repeated. The effects of the isIL-12 construct are shown in A and C; the effects of the isIL-12 construct are shown in b and d. (A, B) Serial tumor measurements were obtained after treatment. N=5 mice per group. Error bars represent the standard error of the mean. P values represent statistical comparisons between the two groups indicated on each graph using matched-pairs, repeated-measures two-way ANOVA tests. (C, D) The change in body weight relative to baseline is shown. Error bars show the standard error of the mean. (E) Serum IL-12 levels and (F) serum IFN-γ levels were determined on day 10 after treatment. The cytokines were measured with a multiplex cytokine kit. Values for individual mice are plotted.; ANOVA, analysis of variance; caIL-12, constitutive anchored interleukin-12; cGFP, constitutively expressed green fluorescent protein; IFN-γ, interferon- γ; isIL-12, inducible secreted interleukin-12.

To assess if the findings observed in the pmel-1 model, which targets a shared tumor-self antigen, were specific to that model, we also assessed caIL-12 in an OT-I model, which targets the foreign antigen, ovalbumin. B16 tumors expressing the ovalbumin-derived peptide, SIINFEKL, (B16-OVA) were treated with OT-I TCR transgenic T cells. Treatment with OT-I cells expressing the previously studied isIL-12 construct improved tumor regression (figure 3A) but also resulted in toxicity as measured by weight loss (figure 3C). In the same experiment, treatment with OT-I cells expressing the caIL-12 construct improved tumor regression (figure 3B). Increasing the number of OT-I cells expressing the caIL-12 construct resulted in greater antitumor activity when the total number of T cells administered remained constant (online supplementary figure S2). The mice cured by OT-I cells expressing either caIL-12 or isIL-12 were protected from rechallenge with B16-OVA tumors (online supplementary figure S3). No weight loss was evident in mice treated with OT-I cells expressing the caIL-12 construct (figure 3D). Consistent with this finding, IL-12 was not detected in the serum of mice after treatment with cells that expressed the caIL-12 construct (figure 3E). IFN-γ was detectable in serum but at lower levels than with the isIL-12 construct (figure 3F). These data indicate that in both pmel-1 and the OT-I tumor models, caIL-12 improved treatment without causing substantial weight loss or elevated serum IL-12 levels.

### Inducible expression of aIL-12 improved tumor treatment in a murine model

**Figure 4:**
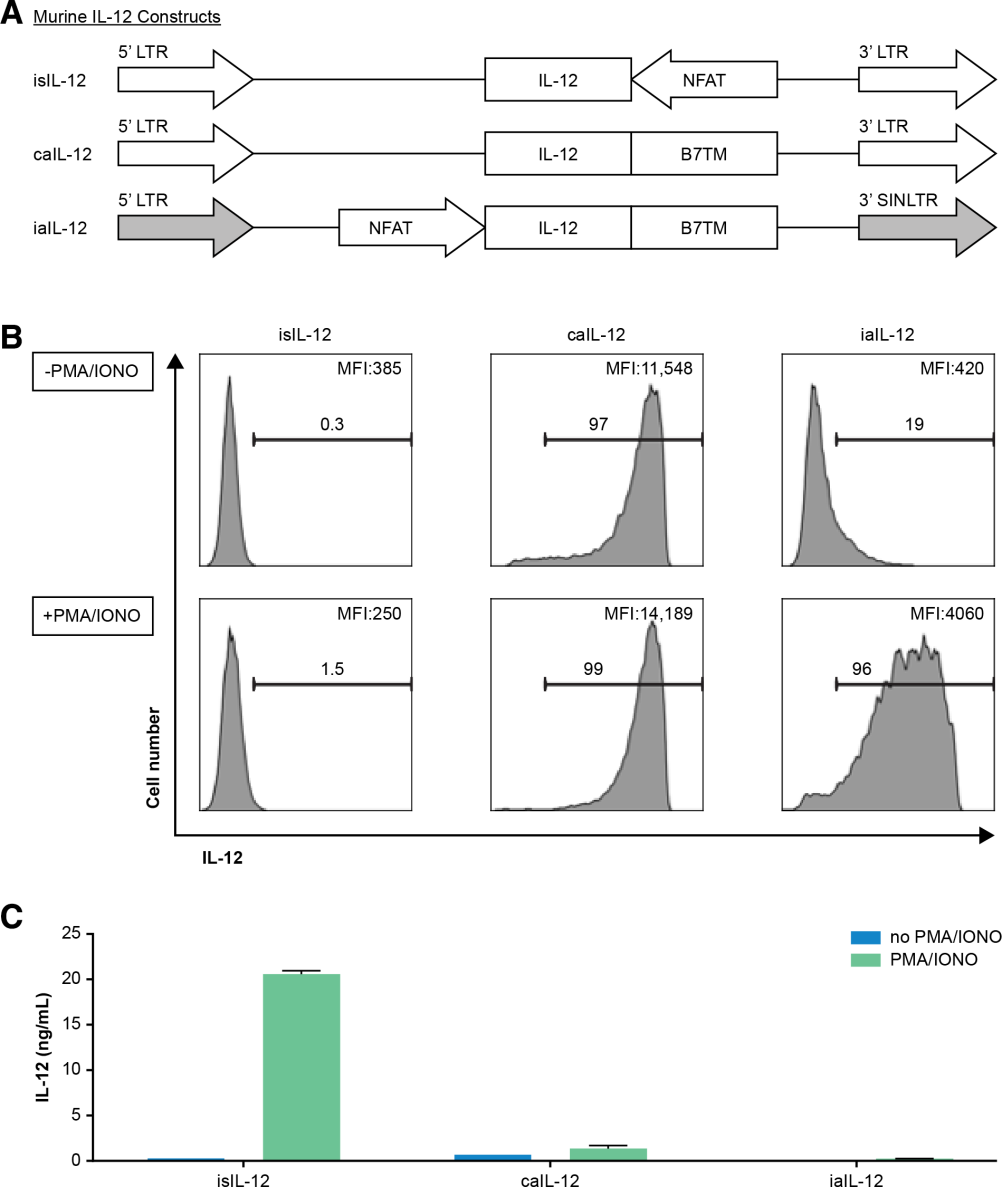
Evaluation of an inducible anchored murine IL-12 construct in vitro. (A) Schematic representation of retroviral vector inserts for murine IL-12 constructs. (B) Human T cells were transduced with the vectors in (A) and cell membrane expression of IL-12 was determined by flow cytometry. Gating is on live cells. Cells were stimulated with PMA/ionomycin overnight as indicated to the left of each row. The frequency of IL-12^+^ cells, and the geometric mean of the fluorescence intensity of IL-12 for each group is indicated on each histogram. (C) Transduced T cells (1×10^6^) were stimulated by PMA/ionomycin and cultured in 1 mL of culture medium overnight, and supernatant IL-12 concentration was determined with an ELISA. caIL-12, constitutive anchored interleukin-12; iaIL-12, inducible anchored interleukin-12; IL-12, interleukin-12; LTR, long-terminal repeat; NFAT, nuclear factor of activated T cells; PMA, phorbol12-myristate 13-acetate.

We sought to determine if aIL-12 could enhance the efficacy of tumor-specific T cells if its expression were constrained by an NFAT promoter. An expression cassette for NFAT-driven transcription of aIL-12 was cloned into a self-inactivating retroviral vector in which the 3’ LTR promoter was deleted (iaIL-12; figure 4A). With this vector, low cell-membrane expression of aIL-12 was observed in the absence of stimulation, and high aIL-12 expression was observed in the presence of stimulation (with PMA and ionomycin), although at lower levels than with caIL-12 (figure 4B). Cells transduced with either the caIL-12 or iaIL-12 vectors displayed low IL-12 shedding (with or without stimulation) based on IL-12 levels in the culture supernatants (figure 4C).

**Figure 5:**
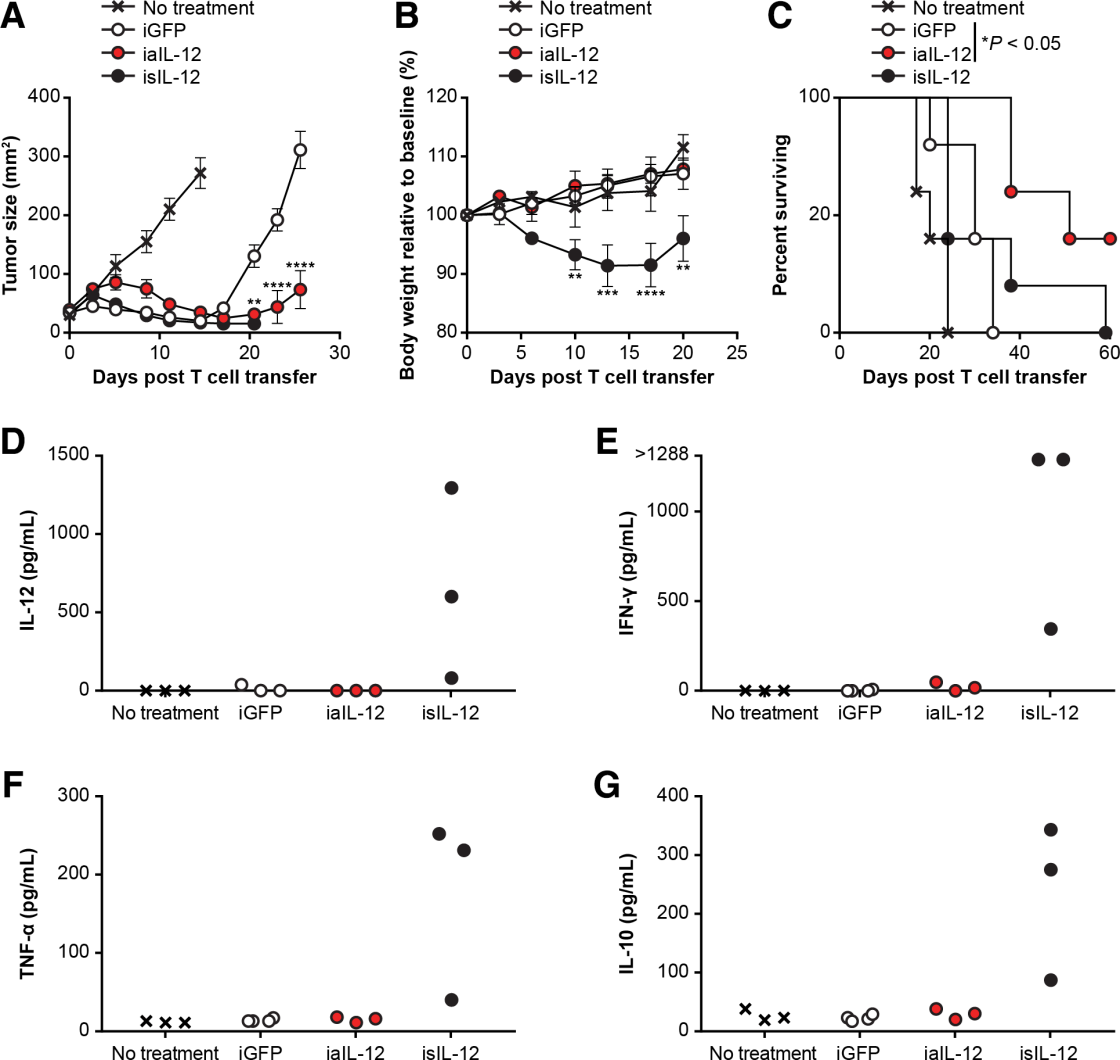
iaIL-12 enhanced the efficacy of tumor-specific T-cell therapy without evidence of toxicity. Mice with established B16-OVA tumors were treated with 15×10^6^ OT-I cells that were transduced with the vectors indicated. isIL-12 served as a positive control for systemic IL-12 exposure. All mice received 550-cGy whole-body radiation before cell infusion. N=5 mice per group. (A) Tumor area was determined by serial measurements at the time points indicated on the x-axis. Error bars represent thestandard error of the mean. P values represent comparisons between iGFP and iaIL-12 at each time point using a two-way ANOVA multiple comparisons test. **P<0.01, ****P<0.0001. (B) The change in body weight relative to baseline is shown. Error bars show the standard error of the mean. P values represent comparisons between iGFP and isIL-12 at each time point using a two-way ANOVA multiple-comparisons test. **P<0.01, ***P<0.001, ****P<0.0001. (C) The survival of tumor-bearing mice that received cell transfer was determined as shown. Survival was greater for iaIL-12 than iGFP (p=0.026 by Mantel-Cox test). (D–G) Serum cytokine levels were determined on day 14 after treatment. The cytokines were measured with a multiplex cytokine kit. Values for individual mice are plotted. The cytokine for each analyte is indicated on the y-axis. isIL-12 serves as a positive control. ANOVA, analysis of variance; iaIL-12, inducible anchored interleukin-12; iGFP, inducible green fluorescent protein; isIL-12, inducible secreted interleukin-12; TNF-α, tumor necrosis factor-α.

To test if iaIL-12 enhances the efficacy of tumor-specific T cells, OT-I cells were transduced with iaIL-12 or control vector (inducible GFP (iGFP)) and administered to B16-OVA tumor-bearing mice. OT-I cells expressing iaIL-12 mediated greater tumor regression than OT-I cells expressing GFP (figure 5A). These mice also did not display weight loss or decreased survival, suggesting that the increased efficacy of iaIL-12 did not come at the expense of increased toxicity (figure 5B, C). Consistent with these findings, iaIL-12 did not cause elevated serum levels of IL-12, IFN-γ, TNF-α, or IL-10 (figure 5D–G).

### aIL-12 enhanced human T-cell–mediated tumor regression in a xenograft model

**Figure 6:**
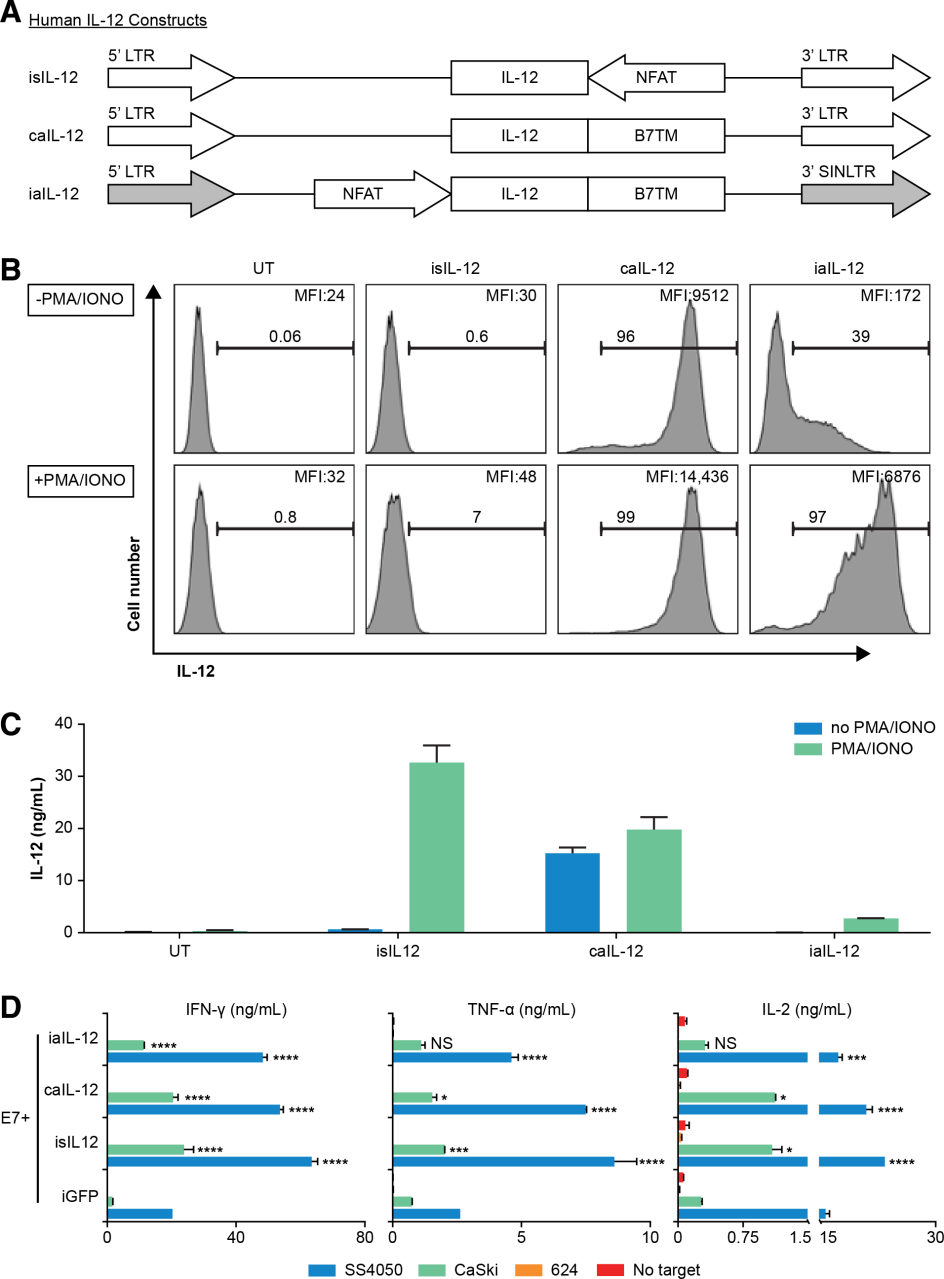
Evaluation of a human iaIL-12 construct in vitro. (A) Schematic representation of retroviral inserts for expression of human IL-12 constructs. (B, C) Human T cells were transduced to express in the construct indicated above each histogram. Two days after transduction, the T cells were activated by PMA/ionomycin overnight. (B) IL-12 cell-surface expression was detected by flow cytometry. Gating is on live lymphocytes. The frequency of IL-12^+^ cells and the geometric mean fluorescence intensity of IL-12 for each group is indicated on each histogram. (C) Transduced T cells (1×10^6^) were cultured in 1 mL of media overnight, and the supernatant IL-12 concentration was determined by an ELISA. (D) Human T cells were cotransduced to express the E7 TCR, and the IL-12 constructs are indicated on y-axis. Five days after transduction, cells were cocultured overnight with tumor cell line targets. SS4050 and CaSki are HLA-A*02:01^+^/E7^+^. 624 is HLA-A*02:01^+^/E7^−^. The concentration of cytokines in the supernatants was determined by an ELISA. Error bars represent the standard error of the mean of two technical replicates. Statistical comparisons were made to the iGFP condition using a two-way ANOVA multiple-comparisons test. *P<0.05, **P<0.01, ***P<0.001, ****P<0.0001. caIL-12, constitutive anchored interleukin-12; E7TCR, T cell receptor targeting human papilloma virus-16 E7; HLA, human leucocyte antigen; iaIL-12, inducible anchored interleukin-12; iGFP, inducible green fluorescent protein; IL-12, interleukin-12; LTR, long-terminal repeat; PMA, phorbol 12-myristate 13-acetate; UT, untransduced cells.

To investigate aIL-12 in human therapeutic T cells, we designed a vector insert encoding a single-chain human IL-12 molecule with a human B7-1 TM anchor and cloned it into retroviral expression vectors (figure 6A). Human T cells transduced to express iaIL-12 showed low cell-membrane IL-12 expression at rest and relatively high cell-membrane IL-12 expression after stimulation with PMA and ionomycin (figure 6B). This finding was evident by both the frequency of cells that expressed IL-12 and the magnitude of IL-12 expression (figure 6B). The concentration of IL-12 in the culture supernatant of iaIL-12–transduced cells was low with or without stimulation, indicating low shedding and/or secretion of the molecule (figure 6C). Expression of membrane aIL-12 could also be induced with CD3/CD28-based stimulation (online supplementary figure S4). We did not test if iaIL-12 could be induced in vitro by antigen-specific T cell stimulation. To investigate the production of effector cytokines by iaIL-12–transduced tumor-specific T cells, human T cells were cotransduced to express a TCR targeting HPV-16 E7 (E7 TCR) and iaIL-12, and the production of cytokines was measured after coculture with target cells. Transduction using iaIL-12 increased the production of IFN-γ, TNF-α, and IL-2 as compared with transduction using negative control (iGFP) when T cells were cocultured with tumor cells expressing the target antigen (SS4050 and CaSki; figure 6D). Cytokine production was not changed by coculture of iaIL-12 transduced with tumor cells not expressing the target antigen (624; figure 6D).

**Figure 7:**
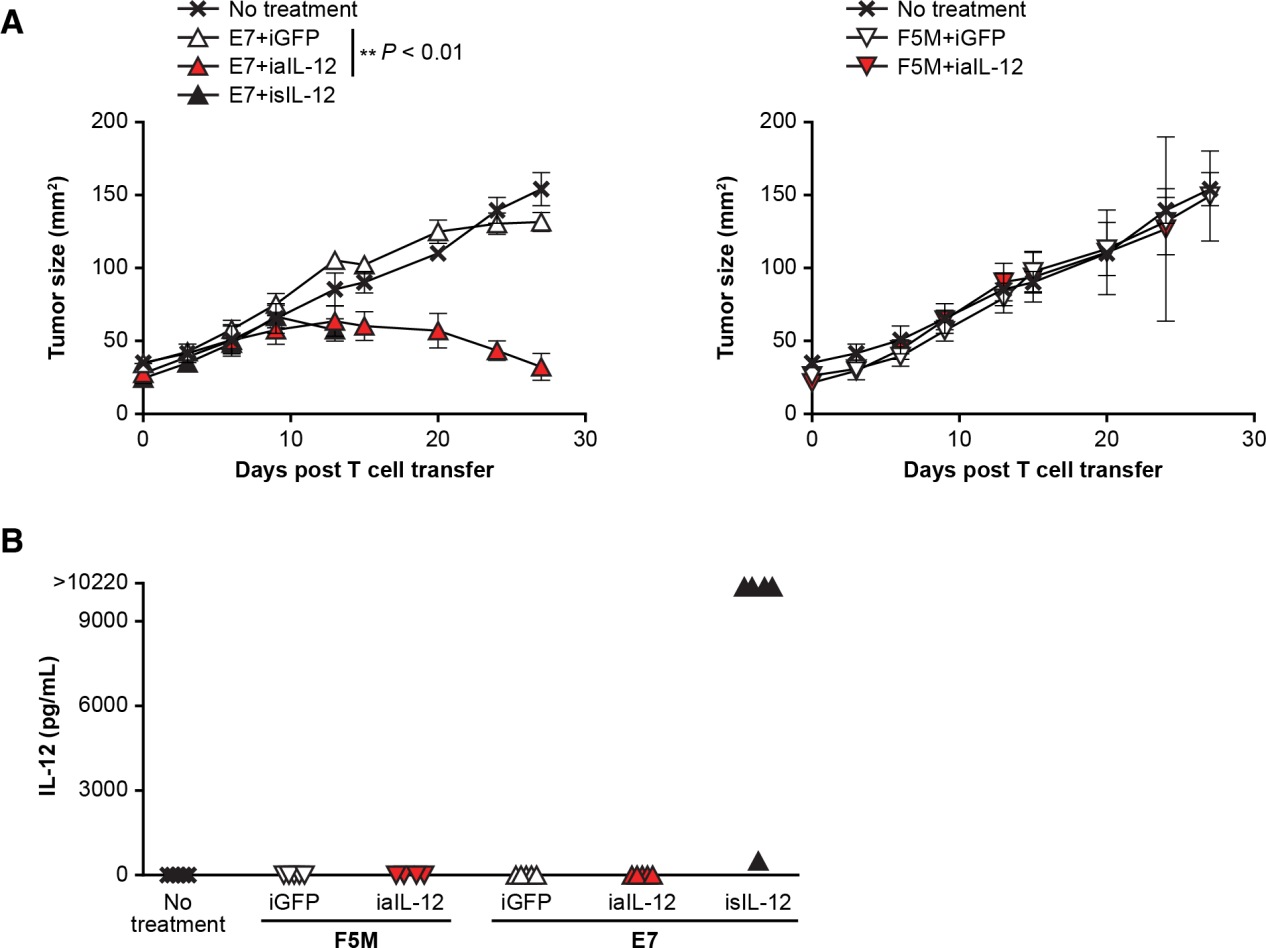
In a human xenograft model, iaIL-12 enhanced treatment efficacy without increasing systemic IL-12 exposure. (A) Human T cells were cotransduced to express the E7 TCR and the IL-12 constructs indicated. The transduced T cells were administered by intraperitoneal injection to immunodeficient mice bearing 12-day, established, CaSki, subcutaneous tumors. isIL-12 served as a positive control for systemic IL-12 exposure. F5M (MART-1-specific TCR) served as a negative TCR control targeting an irrelevant antigen. Serial tumor measurements were obtained. N=5 mice per group. Error bars show the standard error of the mean. P values represent comparisons between iaIL-12 and iGFP with a matched-pairs, repeated-measures two-way ANOVA test. **P<0.01. (B) Serum was collected from treated mice 14 days after treatment. The IL-12 concentration was measured with a multiplex cytokine kit. Values for individual mice are plotted. ANOVA, analysis of variance; E7TCR, T cell receptor targeting human papilloma virus-16 E7; iaIL-12, inducible anchored interleukin-12; iGFP, inducible green fluorescent protein; IL-12, interleukin-12; isIL-12, inducible secreted interleukin-12; TCR, T cell receptor.

To evaluate in vivo tumor treatment with human iaIL-12, the E7 TCR xenograft model was used. In this model, a subcutaneous, established, human cervical cancer that expresses HPV E7 (CaSki) is treated with human E7 TCR gene-engineered T cells (E7 TCR-T cells).^20^ Treatment with E7 TCR-T cells coexpressing iaIL-12 resulted in greater tumor regression than treatment with E7 TCR-T cells coexpressing the negative control (iGFP; figure 7A). Interestingly, treatment with isIL-12 but not with iaIL-12 resulted in high serum levels of IL-12 (figure 7B), and mice treated with isIL-12 but not iaIL-12 died or had to be euthanized after day 15 because of toxicity. Serum levels of IL-12 were not increased in mice treated with iaIL-12 (figure 7B). In summary, in this model, iaIL-12 enhanced treatment efficacy with human tumor-specific T cells and did not increase serum IL-12 levels.

## Discussion

IL-12 is a proinflammatory cytokine with a central role in bridging innate and adaptive immunity. IL-12 has long held promise for cancer immunotherapy; however, thus far, its clinical use has been limited by toxic side effects.^11 12 21^ The data presented here support the safety and efficacy of a new therapeutic delivery system in which IL-12 is preferentially targeted to the tumor via tumor-specific T cells, and IL-12 toxicity is attenuated by restricting its localization to the T-cell surface. One limitation of this study is that toxicity was assessed by body weight and systemic cytokine levels; other toxicology studies were not performed.

Various systems have been proposed to harness the therapeutic potential of IL-12, including (1) tumor-directed delivery (2) regulated IL-12 gene expression, and (3) sequestration of IL-12 to the delivery site. Implementation of these strategies has been facilitated by a fusion protein in which the two IL-12 subunits are linked by a polypeptide connector, permitting expression of IL-12 from a single open reading frame.^18^ In humans, tumor-directed therapy has principally been achieved by intratumoral injection^22–27^ or by T-cell-mediated delivery, as in one clinical trial.^14^ Various methods to improve IL-12-based therapy by controlling gene expression have also been proposed, including the use of an NFAT promoter^16 28^ or a small-molecule gene-activation system.^29^ The advantages of this system include use of a relatively small genetic construct and the absence of a requirement for systemic administration of a gene-activating small molecule. A disadvantage to this system is that the levels of iaIL-12 gene expression are not precisely controlled, and there is a measure of basal iaIL-12 expression (figure 6B). Methods of sequestering IL-12 to the site of delivery have also been investigated. A membrane-anchored IL-12 molecule was previously studied in a model of intratumoral injection of an adenoviral vector.^30^ Another strategy that has been described is deletion of the N-terminal signal peptide to prevent secretion of IL-12 from cells.^31^ In the present study, the B7-1 TM appeared to serve as an effective membrane anchor that limited systemic exposure and toxicity.

The mechanisms by which IL-12 may enhance T-cell–mediated immunotherapy are manifold and not fully understood.^11 12^ IL-12 amplifies the antitumor function of cytotoxic T cells including CD8 T cells, natural killer cells, and natural killer T cells, and it is not clear that recognition of tumor antigens is always required for its effects. The increase in tumor treatment efficacy that results from engineered expression of IL-12 by tumor-specific T cells appears to be due in part to IL-12-mediated effects on bone marrow-derived tumor stromal cells and induction of the Fas death receptor.^32 33^ In the present study in which IL-12 is localized to the T-cell membrane, it is not known which cells IL-12 may influence. IL-12 may act directly on T cells to enhance effector function, and it may signal directly to tumor cells to increase IFN-γ and other cytokines that enhance the immune response. These possibilities are supported by the enhanced antitumor efficacy of iaIL-12 observed in the E7 TCR-T cell model with immunodeficient mice that have widespread defects in endogenous immune cells. IFN-γ is a pleiotropic cytokine. It has demonstrated tumorigenic effects, such as the upregulation of the PDL-1 and PDL-2 checkpoints,^34^ as well as antitumor effects. In tumor-bearing mice, adoptively transferred effector T cells lacking IFN-γ displayed impaired antitumor responses.^35^ IFN-γ may also mediate the antitumor effects of IL-12 and was required for antitumor immunity mediated by antigen-specific T cells expressing secreted IL-12.^32 36^ In our current study, we have demonstrated that in vitro T cells modified by aIL-12 increased IFN-γ production, similar to cells with secreted IL-12, which suggests that these two different forms of IL-12 have similar functions.

It is also possible that aIL-12 may act through bone marrow-derived tumor stromal cells. A limitation of this report was that mechanistic studies were not performed. Experiments in mouse models with intact endogenous immune systems are needed to confirm whether aIL-12 acts mechanistically similar to soluble IL-12,^32 33^ as well as to understand the long-term persistence of aIL-12-modified T cells. In the present study, administration of a smaller number of cells expressing the aIL-12 decreased the efficacy of treatment, even when the total number of therapeutic tumor-specific T cells was held constant. This finding suggests that, if reprogramming of the tumor microenvironment is an important mechanism of action, a small number of cells may not be sufficient to achieve the effect. Additionally, in this study, mice treated with aIL-12 form target-antigen-specific T-cell memory, as they are protected from subsequent tumor rechallenge.

## Conclusion

In summary, the data reported here demonstrate that the antitumor effects of IL-12 can be safely harnessed by the combination of tumor-directed delivery by tumor-specific T cells, T cell-membrane localization, and TCR-signal-induced expression in multiple in vivo tumor models. These data support the testing of iaIL-12 in T-cell therapy for humans. Given the severe toxicities of IL-12 in prior clinical trials, a phase 1 dose-escalation design with careful monitoring of adverse events will be required.
