# Supplementary material for: Enhanced efficacy and limited systemic cytokine exposure with membrane-anchored interleukin-12 T-cell therapy in murine tumor models
Source: J Immunother Cancer. 2020 Jan 19;8(1):e000210. doi: 10.1136/jitc-2019-000210 (PMC7057422; doi:10.1136/jitc-2019-000210)

Zhang, et al. Supplementary Table 1

Table S1. Sequence of transmembrane domains

**Murine B7 TM**	PPEDPPDSKNTLVLFGAGFGAVITVVVIVVIIKCFCKHRSCFRRNEASRETNNSLTFGPEEALAEQTVFL
**Murine CD28 TM**	IEFMYPPPYLDNERSNGTIIHIKEKHLCHTQSSPKLFWALVVVAGVLFCYGLLVTVALCVIWTNSR
**Murine CD8 TM**	TTTKPVLRTPSPVHPTGTSQPQRPEDCRPRGSVKGTGLDFACDIYIWAPLAGICVALLLSLIITLICYHRSR
**Human B7 TM**	DNLLPSWAITLISVNGIFVI CCLTYCFAPR CRERRRNERL RRESVRPV

Zhang, et al. Supplementary Figure 1

Figure S1. Anchored IL-12 improved tumor treatment in a dose dependent manner. Mice with established B16 tumors were treated with pmel-1 cells that were transduced with the vectors indicated. All mice received 550 cGy whole body radiation before cell infusion. Mice received 1×10^5 (**a**), 1×10^6 (**b**) or 5×10^6 (**c**) cells by tail vein injection. Serial tumor measurements were obtained after treatment. N = 5 mice per group. Error bar represent the standard error of the mean. *P*-values represent matched-pair, repeated measure, 2-way ANOVA test between groups indicated in figure legends.

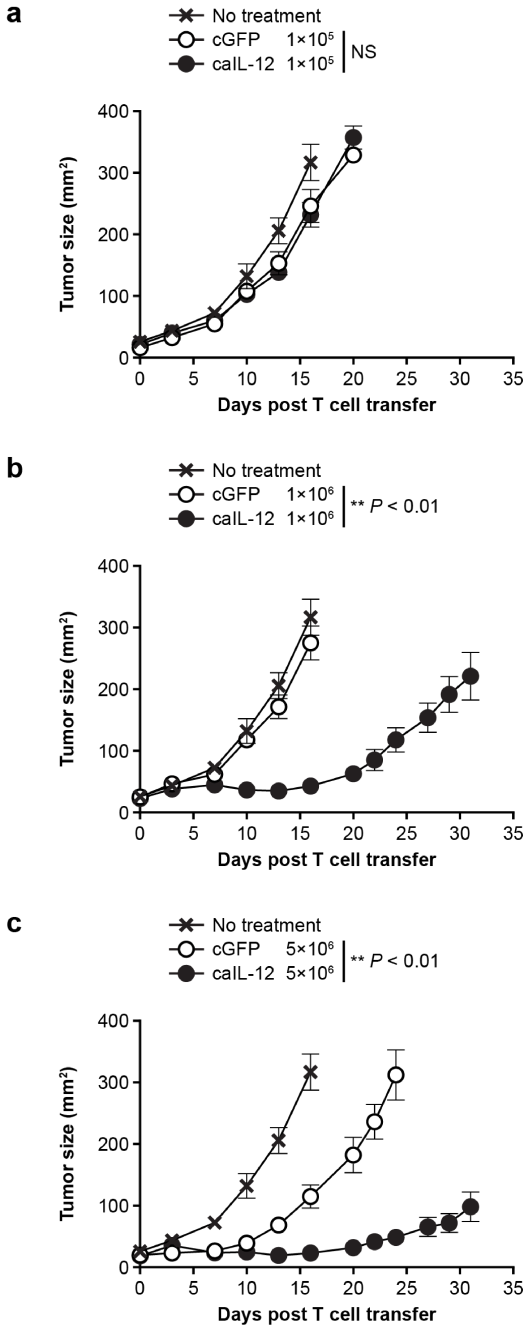

Zhang, et al. Supplementary Figure 2

Figure S2. Increased administration of anchored IL-12 OT-I cells enhanced tumor regression. Mice with established B16-OVA tumors were treated with OT-I cells that were transduced with the vectors indicated. All mice received 550 cGy whole body radiation before cell infusion. Mice received a mixture of caIL-12–modified OT-I cells and non-modified OT-I cells at the ratios indicated. Serial tumor measurements were obtained after treatment. N = 5 mice per group. Error bar represent the standard error of the mean.

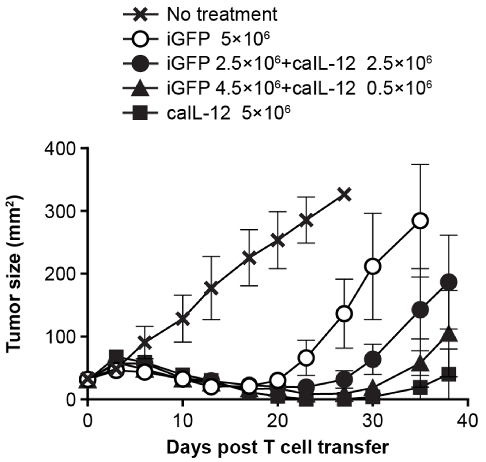

Zhang, et al. Supplementary Figure 3

Figure S3. Anchored IL-12 induction of long-term tumor specific immunity. Mice pretreated by OT-I T cells that were transduced with the vectors indicated in the figure legend or age matched untreated mice (control) were challenged with 1 × 10^6 B16-OVA tumors (**a**) or B16 tumor (**b**) by subcutaneous injection. Serial tumor measurements were obtained after tumor injection. N = 5 mice per group. Error bar represent the standard error of the mean.

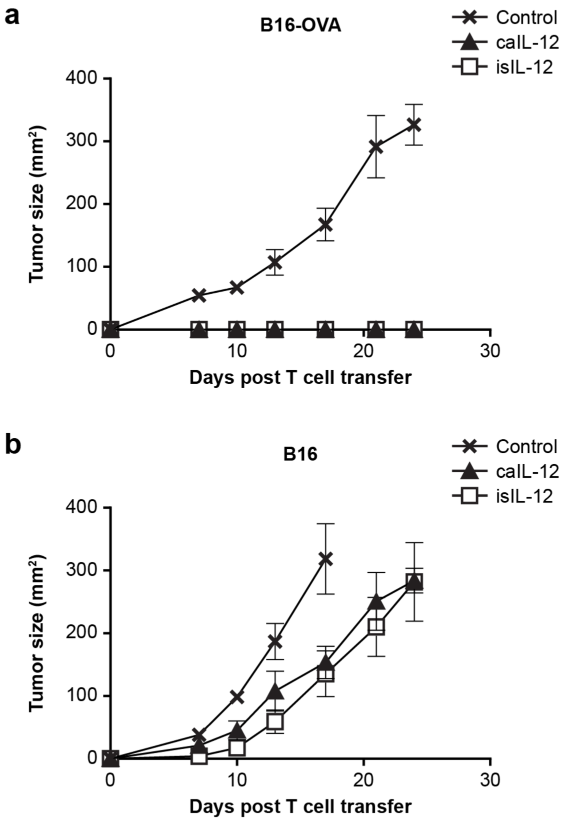

Zhang, et al. Supplementary Figure 4

Figure S4. iaIL-12 was induced by T cell specific activation. Human T cell were transduced to express the construct indicated above each histogram. UT, untransduced cells. Two days post transduction, the T cells were activated by CD3/CD28 Dynabeads at 1:1 ratio for 16 hours. The IL-12 and CD8 surface expression was detected by flow cytometry. Gating is on live lymphocytes. The data represents one of two donors.

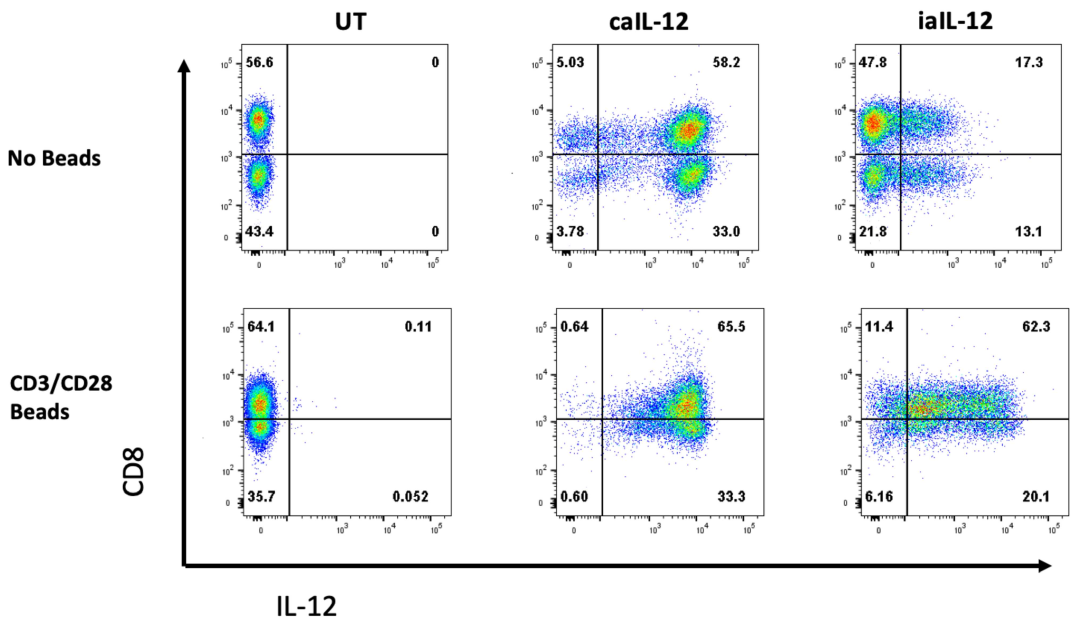

Supplement: Supplementary data [file jitc-2019-000210supp001.pdf]
